# Prediction of adverse health outcomes using an electronic frailty index among nonfrail and prefrail community elders

**DOI:** 10.1186/s12877-023-04160-1

**Published:** 2023-08-07

**Authors:** Kun-Pei Lin, Hsin-Yi Li, Jen-Hau Chen, Feng-Ping Lu, Chiung-Jung Wen, Yi-Chun Chou, Meng-Chen Wu, Ding-Cheng (Derrick) Chan, Yung-Ming Chen

**Affiliations:** 1 Department of Geriatrics and Gerontology, National Taiwan University Hospital, Taipei, Taiwan; 2 Department of Internal Medicine, College of Medicine, National Taiwan University, Taipei, Taiwan; 3 School of Occupational Therapy, College of Medicine, National Taiwan University, Taipei, Taiwan; 4 Medical Department, National Taiwan University Hospital Bei-Hu Branch, No. 87, Neijiang St., Taipei, 108 Taiwan

**Keywords:** Clinical frailty scale, Frailty, Frailty index, Frailty phenotype, Falls, Emergency room visits, Hospitalizations

## Abstract

**Background:**

Early recognition of older people at risk of undesirable clinical outcomes is vital in preventing future disabling conditions. Here, we report the prognostic performance of an electronic frailty index (eFI) in comparison with traditional tools among nonfrail and prefrail community-dwelling older adults. The study is to investigate the predictive utility of a deficit-accumulation eFI in community elders without overt frailty.

**Methods:**

Participants aged 65–80 years with a Clinical Frailty Scale of 1–3 points were recruited and followed for 2 years. The eFI score and Fried’s frailty scale were determined by using a semiautomated platform of self-reported questionnaires and objective measurements which yielded cumulative deficits and physical phenotypes from 80 items of risk variables. Kaplan–Meier method and Cox proportional hazards regression were used to analyze the severity of frailty in relation to adverse outcomes of falls, emergency room (ER) visits and hospitalizations during 2 years’ follow-up.

**Results:**

A total of 427 older adults were evaluated and dichotomized by the median FI score. Two hundred and sixty (60.9%) and 167 (39.1%) elders were stratified into the low- (eFI ≤ 0.075) and the high-risk (eFI > 0.075) groups, respectively. During the follow-up, 77 (47.0%) individuals developed adverse events in the high-risk group, compared with 79 (30.5%) in the low-risk group (*x*^*2*^, p = 0.0006). In multivariable models adjusted for age and sex, the increased risk of all three events combined in the high- vs. low-risk group remained significant (adjusted hazard ratio (aHR) = 3.08, 95% confidence interval (CI): 1.87–5.07). For individual adverse event, the aHRs were 2.20 (CI: 1.44–3.36) for falls; 1.67 (CI: 1.03–2.70) for ER visits; and 2.84 (CI: 1.73–4.67) for hospitalizations. Compared with the traditional tools, the eFI stratification (high- vs. low-risk) showed better predictive performance than either CFS rating (managing well vs. fit to very fit; not discriminative in hospitalizations) or Fried’s scale (prefrail to frail vs. nonfrail; not discriminative in ER visits).

**Conclusion:**

The eFI system is a useful frailty tool which effectively predicts the risk of adverse healthcare outcomes in nonfrail and/or prefrail older adults over a period of 2 years.

**Supplementary Information:**

The online version contains supplementary material available at 10.1186/s12877-023-04160-1.

## Introduction

Frailty is a multidimensional syndrome characterized by increased vulnerability resulting from age-dependent decline in physiologic reserve and homeostatic regulation [1]. Burgeoning studies have shown that frailty is associated with adverse outcomes such as falls [2], hospital (re)admissions, [3, 4] disability [5–7], and all-cause mortality [8]. As world population ageing, it has become a global health burden, with substantial impact for clinical practice and public health [9]. Depending on the operational criteria, study populations and socioeconomic levels, the prevalence of frailty varies greatly from 4–49.3% [10, 11]. Two generally accepted approaches have been used to define frailty, i.e., the rule-based Fried’s frailty scale which measures physical phenotypes [12], and the deficit-accumulation frailty index (FI) which quantitates health vulnerability [13]. A third class of approach, the judgement-based Clinical Frailty Scale (CFS), was developed as a handier tool to measure fitness and frailty in older adults [14], and has since been associated with outcomes in multiple clinical settings [15]. In Rockwood et al’s original cohort, elders with CFS 4–7 points were more likely to die and enter an institution than those with CFS 1–3 points. The outcome of individuals graded as ‘managing well’ to ‘fit’ (CFS 2–3 points) [16], while not directly compared to the ‘very fit’persons (CFS 1 point), could be deduced from Fried’s model where subjects with ‘intermediate or prefrail status’ (1–2 criteria) were found to exhibit worse outcomes than their ‘nonfrail’ (0 criteria) counterparts [12].

Nonfrail and prefrail individuals represent the vast majority of community-dwelling older adults. Nonfrail elders especially those without prior major health events are generally considered as ‘fit to very fit’ or ‘robust’. However, in a recent systematic review [17] pooling 120,805 nonfrail older adults from 46 observational studies, the incidence rate of prefrailty was much higher than frailty (about 151 new cases of prefrailty per 1000 person-years vs. 43 new cases of frailty). Prefrailty, like frailty syndrome mentioned above, is associated with adverse health outcomes [11, 17]. Thus, it is imperative to identify susceptible elders so that disabling conditions can be preventive by timely instructions or interventions [18–23].

Given the subtleness of health changes in nonfrail and prefrail older adults, we hypothesized that the deficit-accumulation FI approach, which was rooted on the concept of comprehensive geriatric assessment (CGA) [24], may detect health vulnerability and predict adverse outcomes more extensively than traditional rule-based tools [2, 25–27]. However, the procedure of data acquisition from any given FI is often time-consuming and unwieldy in clinical practice [9, 28]. As such, assessment by aid of routinely available electronic health record data has been used to form FIs to expedite frailty screening [29–31]. While these FIs identified susceptible elders with descent predictive validity, the electronic medical records lacked essential elements of physical phenotypes such as grip strength and walking speed, which failed to enable individually tailored preventive actions. Newer digital devices have also been adapted to measure frailty components such as walking speed and gait [32, 33], yet most instruments used were stand-alone and not interconnected. Here, we report the usefulness of a semiautomated electronic FI (eFI) system which comprised 80 risk factors of health deficits. The predictive performance of the eFI was analyzed and compared to that achieved with the traditional CFS and Fried’s frailty scale in a prospective cohort of nonfrail and prefrail community elders followed over a period of 2 years.

## Materials and methods

### Study design and setting

This prospective cohort study was conducted at the Department of Geriatrics and Gerontology of a tertiary medical center. Community-dwelling older people who received geriatric health examinations were recruited from April 2018 to December 2018. The study protocol was reviewed and approved by the Research Ethics Committee of the National Taiwan University Hospital (No: 201802035RINB).

### Participants

Older adults aged 65–80 years with basic literacy skills and a CFS rating of 1–3 points using a validated traditional Chinese version [34] were enrolled and followed for 2 years. Patients with dementia or active cancer and those who were unable to follow measuring instructions were excluded. Individuals with pacemakers or metal implants were excluded to avoid interference using the bioelectrical impedance analysis. Formal written informed consent was obtained from each individual before participating in this study.

### Assessment of frailty risk

The severity of frailty was assessed by using an 80-item eFI built in the BabyBot vital data recording system (Netown Corporation, Taipei, Taiwan), which yielded the deficit-accumulation eFI score and the Fried’s frailty phenotype.

### Deficit-accumulation eFI score

The eFI system used a count of 80 ‘health deficits’ (risk factors) whose selection were in accord with the criteria of construction and ascertained by an expert team of geriatricians listed as authors on this paper. The full list of variables is provided in **Supplementary Table**. Among these factors, 68 subjective items were obtained by self-reported questionnaires presented on a touchscreen tablet interface, while 12 objective items were measured using medical devices approved by Taiwan Ministry of Health and Welfare, including a three-in-one machine (OMRON Automatic Blood Pressure Monitor; BabyBot Pulse Oximeter) for vital signs, a bioelectrical impedance analyzer (Tanita BC-418, Tokyo, Japan) for body composition and body mass index, a hand-held dynamometer with digital output for hand grip strength, a Gaitspeedometers with infrared sensors devices for walking speed, as well as a cushion-type pressure sensor for timed up and go test and 5 times sit-to-stand test. The assessment of each participant was conducted under the guidance of a trained assistant. The reply to questionnaires and the results of measurements were uploaded to the internet without manual recording (**Supplementary Figure**). The eFI system assigns equal weights to all 80 included items. One point was given for each abnormal deficit, and the cumulative deficit (range 0–80) was translated into the eFI score by calculating the sum of all deficits, divided by the total 80 risk factors included in the system (eFI score = 0–1). Given the nature of risk factors included, and to simplify the interpretation of results, we chose a cutpoint based on the median eFI score. Individuals with an eFI score ≤ the median value were defined as ‘low risk’, while those with an eFI score > the median were classified as ‘high risk’. To confirm the predictive accuracy of the categorical classification, a separate Cox regression model was constructed, treating the eFI score from 0 to 1 as continuous variable.

### Fried’s frailty phenotype

The rule-based frailty phenotype is defined according to the following 5 criteria: unintentional weight loss (5 kg in the past year), self-reported exhaustion, weakness (grip strength), slow walking speed, and low physical activity. The result of each criteria was extracted during the same round of assessment in obtaining the eFI score. Individuals with a frailty score of 0, 1–2 and > 2 are classified into nonfrail, prefrail and frail groups, respectively.

### Outcome measures

During the 2-year follow-up, any incident adverse events including falls, emergency room (ER) visits and unexpected hospitalizations, were collected every three months through telephone interviews. A fall episode was defined by the WHO as ‘an event which results in a person coming to rest inadvertently on the ground or floor or other lower level.’

### Statistical analyses

SAS version 9.4 (SAS Institute, Cary, NC) was used for analyses. T tests (for normally distributed continuous variables), Mann–Whitney U tests (for nonnormally distributed continuous variables) and chi-square tests (for categorical variables) were used for between-group comparisons. Curves for the probability of falls, ER visits and hospitalizations within 24 months were created with the Kaplan–Meier method and compared using the log-rank test. Multivariate analysis adjusted for age and sex was performed using a Cox proportional hazards regression model to project the impact of frailty risk (high- vs. low-risk, as categorical variable) or eFI score (0–1, as continuous variable) on adverse health outcomes of falls, ER visits and hospitalizations. A p value < 0.05 was considered significant.

## Results

### Approaches of utilizing the 80-item eFI system

A total of 427 older adults with a CFS of 1–3 points underwent evaluation by the eFI system on the index date. For pragmatic reasons, we divided participants into groups of one or more (2 to 7) people. Ten persons were assessed in one-by-one approach, 34 in groups of 2 (17 groups), 84 in groups of 3 (28 groups), 112 in groups of 4 (28 groups), 130 in groups of 5 (26 groups), 36 in groups of 6 (6 groups) and 21 in groups of 7 (3 groups). As shown in Table 1, in one-by-one evaluation approach, the assessment was completed in 18.1 min on average. In group evaluation approach, it took an average of 19.6 min for 2 people, 20.7 min for 3 people, 22.8 min for 4 people, 24.8 min for 5 people, 31.9 min for 6 people and 39.7 min for 7 people. The more participants each group contained, the longer operation time it took to complete the whole assessment. That being said, group approach was more time-efficient than one-by-one approach. We found that groups of ≧ 3 persons could save up to 60% of the estimated total time with one-by-one approach.

**Table 1:** Time efficiency in utilizing the eFI system

	One-by-one approach	Group approach					
Number of person per group	1	2	3	4	5	6	7
Estimated time spent, minutes	18.1	36.2	54.3	72.4	90.5	108.6	126.7
Actual time spent, minutes (mean ± SD)	18.1 ± 1.2	19.6 ± 1.0	20.7 ± 0.9	22.8 ± 1.2	24.8 ± 1.0	31.9 ± 1.9	39.7 ± 1.5
Time saved (vs. one-by-one approach), minutes	NA	47%	62%	69%	73%	71%	69%

eFI, electronic frailty index; SD, standard deviation; NA, not applicable

### Baseline characteristics of the participants

**Fig. 1:**
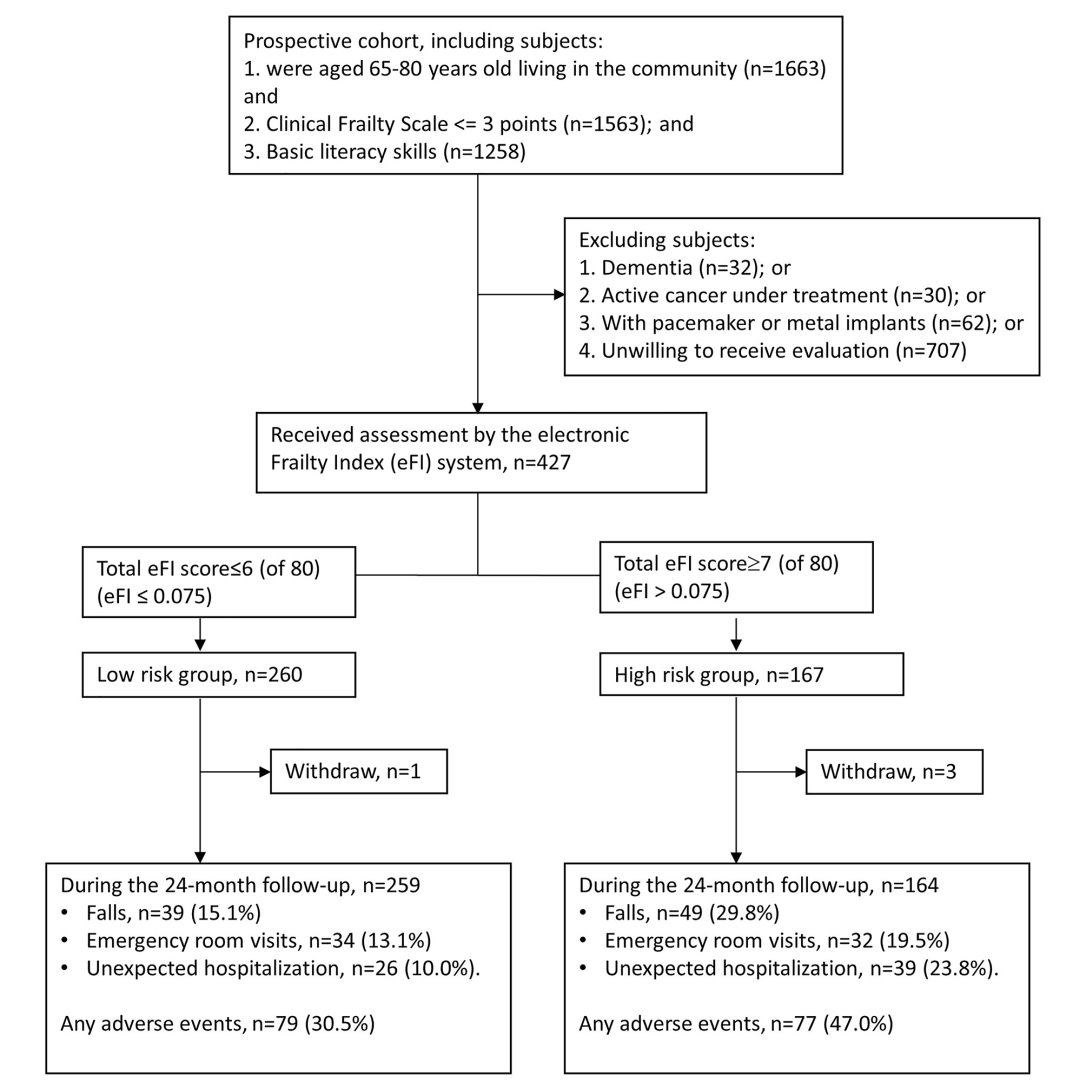
Flowchart of the study

The mean age of the participants was 71.3 years, with 197 (46.1%) being men. The median eFI score was 0.075 with an interquartile range of 0.0625. Two hundred and sixty elders were categorized as low-risk (i.e., eFI score ≤ 0.075), while 167 were classified as high-risk (i.e., eFI score > 0.075) Four participants (1 low-risk, 3 high-risk) withdrew voluntarily shortly after the initial assessment due to personal reasons. (Fig. 1). The baseline demographics, clinical characteristics and functional status of the two groups are shown in Table 2. No significant differences in sex, age, marital status or education level were observed. In men but not women, the high-risk group exhibited significantly lower grip strength than the low-risk group (29.3 kg vs. 31.9 kg, p = 0.003). The high-risk group also reported more medical conditions, including hypertension, diabetes mellitus, hyperlipidemia, coronary artery disease, chronic obstructive pulmonary disease, chronic liver disease and urologic disorders. The average eFI score of the high- and the low-risk groups was 0.11 and 0.05, respectively.

**Table 2:** Baseline demographics, clinical characteristics and eFI score of the participants

	All participants, n = 427	Low-risk group, n = 260	High-risk group, n = 167	p value
Men, no.(%)	197 (46.1)	121 (46.5)	76 (45.5)	0.83
Age, years, mean (SD)	71.3 ± 4.1	71.1 ± 4.0	71.5 ± 4.1	0.33
Marital status, no.(%)				
Divorced, widowed, or single	52 (22.7)	52 (20.0)	45 (26.7)	0.09
Education, no.(%)				
College/University and above	320 (74.9)	198 (76.0)	122 (73.1)	0.47
Grip strength, kg, mean (SD)				
Men	30.9 (5.9)	31.9 (5.9)	29.3 (5.4)	0.003
Women	19.5 (3.2)	19.8 (3.2)	19.2 (3.2)	0.14
Walking speed, m/s, mean (SD)	1.35 (0.3)	1.36 (0.3)	1.32 (0.3)	0.08
Body mass index, kg/m^2^ (SD)	23.6 (3.1)	23.2 (2.6)	24.1 (3.7)	0.003
Metabolic syndrome, no.(%)	97 (22.7)	40 (15.4)	51 (34.1)	< 0.0001
Comorbidities, no.(%)				
Hypertension	149 (34.9)	67 (25.7)	82 (49.1)	0.0004
Diabetes mellitus	41 (9.6)	16 (6.2)	25 (15.0)	0.003
Hyperlipidemia	110 (25.8)	45 (17.3)	65 (38.9)	< 0.0001
Coronary artery diseases	62 (14.5)	19 (7.2)	43 (25.8)	< 0.0001
Chronic obstructive pulmonary disease	20 (4.7)	5 (1.9)	15 (9.0)	0.0008
Chronic liver diseases	25 (5.9)	9 (3.5)	16 (9.6)	0.0009
Urologic diseases	38 (8.9)	13 (5.0)	25 (15.0)	0.0004
eFI score, median (IQR)	0.075 (0.0625)	0.05 (0.025)	0.1125 (0.05)	< 0.0001

eFI, electronic frailty index; IQR, interquartile range; SD, standard deviation

Adverse events predicted by the 80-item eFI score and the traditional tools.

As shown in Table 3, the eFI scoring system identified 260 (60.9%) individuals as low-risk. In contrast, CFS graded 83.4% of the participants as very fit (4.0%) to well (79.4%), while Fried’s frailty scale reported 281 (65.8%) individuals were robust and 144 (33.7%) were prefrail. Despite the discrepancies in the classification, the trends of frailty risks as revealed by different frailty tools remained statistically significant (Spearman correlation tests, p < 0.0001). It is noteworthy that among the ‘relatively healthy’ elders, defined by either very fit to fit with CFS or robust with Fried’s scale, approximately one-third of them (34.8% and 30.2%, respectively) were stratified as high-risk by the eFI scoring system. Moreover, up to 60.6% of managing-well individuals by CFS rating and 55.6% of prefrail individuals by Fried’s scale were stratified as high-risk with the eFI system. These data suggest that the eFI system provided more discriminative evaluation of overall health deficits among individuals with a CFS score of 1–3 points or Fried’s scale of 0–2 criteria.

**Table 3:** Correlations between different risk levels classified by eFI, CFS, and Fried’s scale

	eFI category Low risk, n = 260 (60.9%)	eFI category High risk, n = 167 (39.1%)	p value^#^
Clinical Frailty Scale			
1 (very fit)	16 (94.1%)	1 (5.9%)	< 0.0001*
2 (fit)	216 (63.7%)	123 (36.3%)	
3 (managing well)	28 (39.4%)	43 (60.6%)	
Fried’s frailty scale			
Nonfrail	196 (69.7%)	85 (30.3%)	< 0.0001*
Prefrail	64 (44.4%)	80 (55.6%)	
Frail	0 (0.0%)	2 (100%)	

^#^Statistics done by the Spearman correlation test

**Fig. 2:**
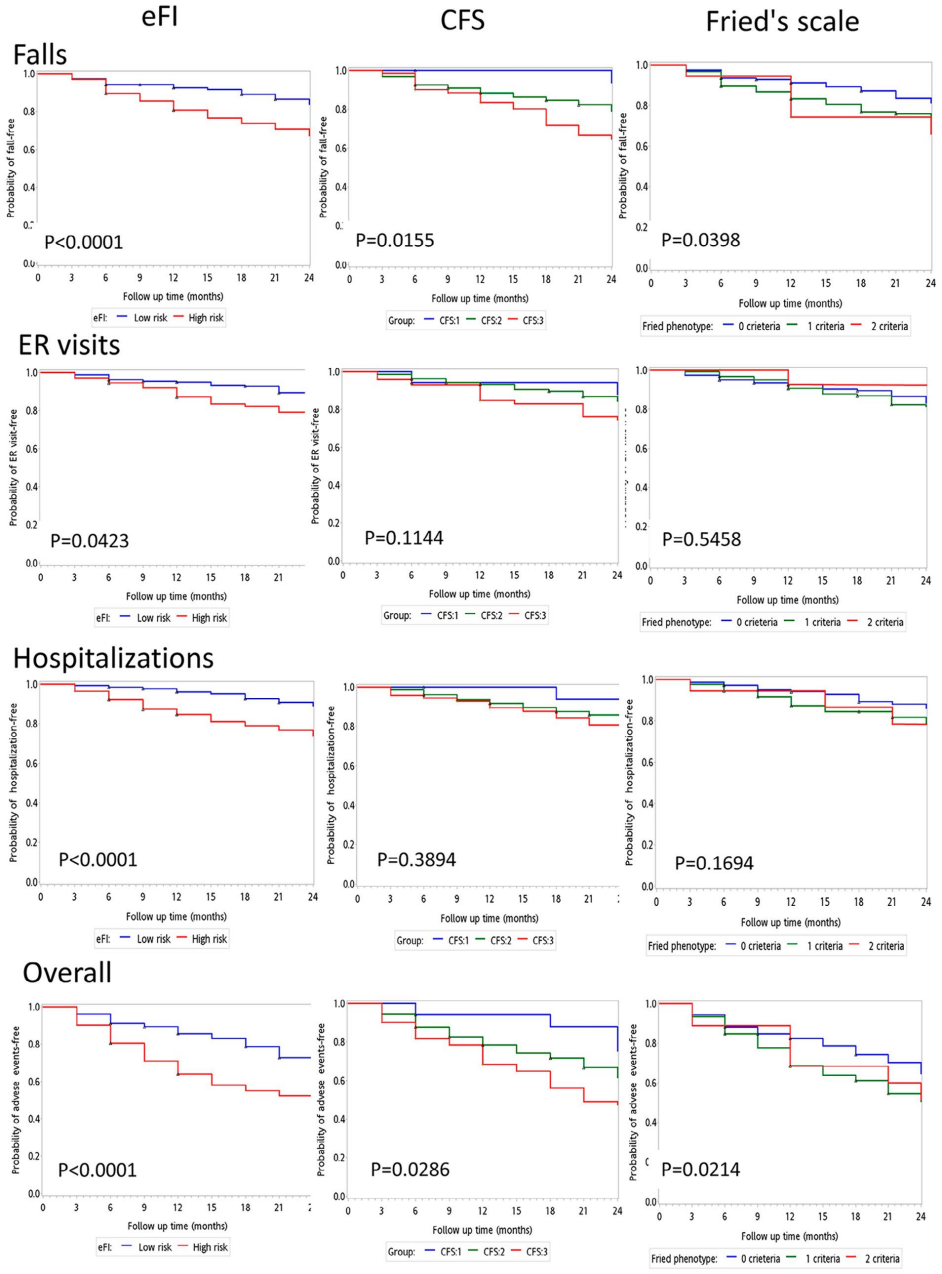
The event-free survival curves of falls, ER visits and hospitalizations between the high- and low-risk groups stratified by the eFI system, as well as subsets classified by CFS rating (1,2,3 points) and Fried’s frailty scale (0, 1, 2 criteria)

During the 2-year follow-up, 77 of 164 (47.0%) high-risk participants and 79 of 259 (30.5%) low-risk elders experienced either of the three adverse events (*x*^*2*^, p = 0.0006). The Kaplan–Meier analysis shows that the event-free survival curves of falls, hospitalizations, and ER visits during the 24-month follow-up were significantly better in the low-risk than in the high-risk group (log-rank test, p < 0.0001 for falls, p = 0.04 for ER visits, p < 0.0001 for hospitalizations) In contrast, the survival curves graded by CFS scores (1,2,3 points) and Fried’s scale (0, 1, 2 criteria) were less discriminative compared with that achieved by the eFI stratification (high- vs. low-risk) (Fig. 2).

In the multivariable models adjusted for age and sex (Table 4), the high-risk elders were associated with an increased probability of adverse health outcomes than their low-risk counterparts (adjusted hazard ratio (aHR) (95% confidence interval (CI)) = 2.20 (1.44–3.36) for falls; 1.67 (1.03–2.70) for ER visits; 2.84 (1.73–4.67) for hospitalizations; and 3.08 (1.87–5.07) for all three events combined). These trends remained the same even if the eFI score from 0 to 1 was treated as continuous variable (1.09 (1.06–1.13); 1.07 (1.03–1.13); 1.09 (1.05–1.14); 1.11 (1.07–1.16) for falls, ER visits, hospitalizations, and all three events combined, respectively). Compared with the traditional tools, the 80-item eFI classification, i.e., high- vs. low-risk, performed better regarding the prediction of adverse health outcomes than did either CFS rating (managing-well vs. fit to very fit), not discriminative in hospitalizations) or Fried’s scale (prefrail to frail vs. robust, not discriminative in ER visits).

**Table 4:** Hazard ratios for adverse events by different frailty-assessing tools

	Falls	ER visits	Hospitalizations	Overall adverse events
eFI score (by category)				
Low Risk, n/total (%)	39/259(15.1)	34/259(13.1)	26/259(10.0)	79/259(30.5)
High Risk, n/total (%)	49/164(29.8)	32/164(19.5)	39/164(23.8)	77/164(47.0)
Adjusted HR (95% CI)	2.20(1.44–3.36)*	1.67(1.03–2.70)*	2.84(1.73–4.67)*	3.08(1.87–5.07)*
p value	0.0002	0.039	< 0.0001	< 0.0001
**eFI score (continuous variable)**				
Adjusted HR (95% CI)	1.09(1.06–1.13)*	1.07(1.03–1.13)*	1.09(1.05–1.14)*	1.11(1.07–1.16)*
p value	< 0.0001	0.001	< 0.0001	< 0.0001
**CFS score**				
1–2 points, n/total (%)	66/352(18.8)	50/352(14.2)	51/352(14.5)	123/352(34.9)
3 points, n/total (%)	22/71(31.0)	16/71(22.5)	14/71(19.7)	33/71(46.5)
Adjusted HR (95% CI)	1.69(1.03–2.75)*	1.97(1.11–3.50)*	1.70(0.93–3.09)	1.59(1.07–2.35)*
p value	0.0368	0.0209	0.0849	0.0209
**Fried’s frailty scale**				
Nonfrail, n/total (%)	48/279(17.2)	43/279(15.4)	36/279(12.9)	91/279(32.6)
Prefrail to frail, n/total (%)	40/144(27.8)	23/144(16.0)	29/144(20.1)	65/144(45.1)
Adjusted HR (95% CI)	1.66(1.08–2.53)*	1.15(0.69–1.92)	1.94(1.18–3.21)*	1.60(1.16–2.22)*
p value	0.0203	0.6021	0.0091	0.0045

h, hazard ratio; ER, emergency room; CI, confidence interval; CFS, Clinical Frailty Scale, eFI, electronic frailty index. *p < 0.05

## Discussion

This prospective observational study demonstrated for the first time that a novel, semiautomated eFI system effectively predicted the risk of adverse health outcomes among a cohort of nonfrail and prefrail community elders followed over a period of 2 years. Our participants were recruited based on CFS rating from very fit (CFS 1 point) to managing well (CFS 3 points), with Fried’s phenotype ranging from 0 to 1–2 criteria, and a median eFI score of 0.075 (interquartile range 0.0625), which all fit the operational definitions of nonfrailty and/or prefrailty [9, 12, 16]. The outcome of individuals graded as ‘managing well’ to ‘fit’ (CFS 2–3 points) [16] might be comparable to prefrail elders (Fried’s scale 1–2 criteria) who showed intermediate risk of incident falls, worsening mobility or disabled activity of daily living, hospitalization, and death [12]. The present study further found that elders with an eFI score > 0.075, i.e., the high-risk group, displayed an increased risk of falls, ER visits and hospital admissions, compared with their low-risk counterparts. More importantly, in multivariable models adjusted for age and sex, the overall predictive performance of the eFI stratification (high- vs. low-risk) was more discriminative than that projected by either CFS rating (CFS 3 vs. CFS 1–2 points) or Fried’s scale (prefrail to frail vs. robust). These findings may have implications for practicing physicians in terms of identifying susceptible individuals, deploying preventive actions, and allocating healthcare resources. Indeed, a plethora of studies have shown that multidomain and interdisciplinary primary care interventions can reverse prefrailty to robustness among prefrail older adults [18–21].

Community-dwelling older adults, whether nonfrail or prefrail, are prone to developing frailty as they get older [17, 35]. It is also possible that due to reduced physiological reserve or intrinsic capacity, some elders may become frail and enter into disability prematurely following inadvertent adverse events such as falls or hosptalizations. Thus, early detection of susceptible individuals at greater risk of adverse health outcomes is of paramount importance so that frailty progression and its consequential outcomes can be prevented [23]. Among the traditional tools, CFS is a handy clinical index and Fried’s phenotype is a brief and concise scale, both of which have been used for screening and prediction since their launch. However, CFS is judgement-based requiring experienced physicians to maintain interrater reliability, while Fried’s phenotype is rule-based focused mainly on physical domains without referring to cognitive or psychosocial dimensions [36]. By comparison, the CGA-based FI model [24] has shown better or non-inferiority discriminative power in risk identification and outcome prediction [2, 25–27]. But the time-consuming and unwieldy nature of this approach prevents its routine use in daily practice [9, 28].

The present study provided an alternative solution to this problem by demonstrating the implementation of a semiautomated eFI system in nonfrail and prefrail community elders. It is noteworthy that approximately 60% of managing-well seniors (CFS 3 points) or prefrail elders by Fried’s phenotype (1–2 criteria) were stratified as high-risk, suggesting that the eFI system might be more discriminative than traditional tools in evaluating the health status of community elders. Technically, the most efficient way of utilizing the system was to adopt the ‘team’ approach. Our data showed that groups of 3–5 individuals could allow a trained assistant to complete a full screening, saving up to 60% of the estimated total time compared with the ‘one-by-one’ approach. The built-in 80 risk variables also contained elements of Fried’s phenotypes (walking speed, grip strength) and the Study of Osteoporotic Fractures index (5 time sit-to-stand) [37], which could be measured during the same round of eFI assessment. This add-on value makes the eFI system a very useful tool to screen for frailty status particularly in ‘subhealthy’ older adults residing in the community.

This study is unique in several aspects. First, the 80 items of health deficits included in the system could be obtained in real time without any missing data, thus increasing the reliability of its predictive utility. Second, the platform of eFI can be operated in a semiautomated manner by a single person, which greatly reduces the unwieldy nature of calculating FI scores. Third, the eFI system contains all the necessary elements to measure frailty risk defined by other traditional tools such as Fried’s frailty scale and SOF, making it a handy tool to screen for frailty in older adults. Last but not least, compared to CFS score and Fried’s scale, the eFI system provided more discriminative power in predicting adverse outcomes. This add-on benefit may help the clinicians or medical professionals to deliver more individually tailored preventive actions so that future disability can be averted.

As a new technology-based tool, there are some limitation and uncertainties about the eFI system. First, the self-reported questionnaires as displayed on touchscreen tablet interface might be subject to reporting bias. Nevertheless, self-reported tools such as Kihon Checklist and FRAIL are widely used for frailty screening [38, 39], and our participants could complete the current touchscreen survey under the assistance of a trained person, which substantially reduced any inconsistency during the evaluation. Second, some studies proposed cutoffs > 0.25 to indicate frailty, and 0.1 to 0.25 as prefrailty [9, 40]. In this study, we used the median eFI score of 0.075 to stratify our nonfrail and prefrail participants. Although this may seem arbitrary, we also constructed a separate Cox regression model, treating the eFI score from 0 to 1 as continuous data, and the results remained consistent with that projected by the categorical stratification. Finally, because three outcomes (fall, ER visit and hospitalization) were set to evaluate the predictive utility of the eFI, CFS, and Fried’s scale, type 1 error might arise due to multiple testing. To adjust for this potential bias, we conducted Bonferroni correction by multiplying the p-values by a factor of three in Table 4, and the results confirmed that the eFI scores, especially when treated as continuous variable, remained a significant tool in the prediction of all outcomes measured.

## Conclusions and implications

The 80-item eFI system effectively predicts the risk of adverse health outcomes in a prospective cohort of nonfrail and prefrail community elders during a 2-year period of follow-up. Compared to CFS score and Fried’s scale, the eFI system provided more discriminative power in predicting adverse outcomes. These findings may have implications for practicing physicians in terms of identifying susceptible individuals, deploying preventive actions, and allocating precious healthcare resources. More information is needed to prove its application in other clinical settings, and its usefulness in outcome protection by provision of healthcare instructions or interventions.

### Electronic supplementary material

Below is the link to the electronic supplementary material.

Supplementary Material 1

Supplementary Material 2

## Data Availability

The data that support the findings of this prospective, observational study are not openly available but are available from the corresponding author upon reasonable request. The data presented here are available within the article or supplementary materials.
