# Supplementary material for: Prediction of adverse health outcomes using an electronic frailty index among nonfrail and prefrail community elders
Source: BMC Geriatr. 2023 Aug 7;23:474. doi: 10.1186/s12877-023-04160-1 (PMC10408173; doi:10.1186/s12877-023-04160-1)
Supplement: Supplementary file 1 — Supplementary Material 1 [file 12877_2023_4160_MOESM1_ESM.doc]

**Supplementary Table.** List of variables used to construct the 80-item electronic frailty index

| Domains | Risk factors (deficits) |
| --- | --- |
| Cognition and affect  (17 items) | Education level≦primary school graduate; unable to make medical decisions by oneself; no religious belief; trouble with short-term memory; difficulty in learning to use new tools; difficulty in handling daily affairs; problems for judgment making; unable to recall correct month or year; feel unhappy most of the time; loss of interests; feeling life is empty; feeling situation is hopeless; extreme fear of death; fear to discuss advance healthcare directive; unable to accept life changes after retirement; unable to accept changes by aging; unwilling to seek for medical assistance |
| Comorbidity  (19 items) | Hypertension; diabetes mellitus; hyperlipidemia; cerebrovascular disease; cardiovascular disease; chronic obstructive pulmonary disease; chronic hepatitis; urologic disease; cancer; sleep disorder with need of medication; neurodegenerative disease; thyroid disorder; chronic gastrointestinal disease; anemia; osteoarthritis; osteoporosis; spinal disorder; gout; autoimmune disease |
| Nutrition and physiology  (13 items) | Regular smoking; regular alcohol consumption; lack of regular exercise; nutrition disorder; body weight loss >5% or 3 kg in the past year; missing teeth affecting chewing and eating; abnormal systolic blood pressure; abnormal diastolic blood pressure; abnormal pulse rate; abnormal SpO_2_; abnormal waist circumference; abnormal body mass index; abnormal body fat proportion |
| Fall risk  (18 items) | Living alone; living upstairs without an elevator; no one can help when needed; low physical activity; fall history in recent one year; feeling comfortable for the past month; need to use a cane for ambulation; poor balance or frequent dizzy spells; easy fatigability; difficulty in walking for more than 100 meters; difficulty climbing more than 10 steps; multiple clinic visiting; polypharmacy; abnormal grip strength; abnormal test of timed up and go; abnormal walking speed; abnormal skeletal muscle mass index; abnormal five times sit-to-stand test |
| Activity and communication  (13 items) | Difficulty for communication; need help for dressing; need help for eating; need help for moving; poor bladder or bowel control; unable to take a bath alone; unable to shop alone; unable to do household chores; incapable of handling money; having trouble to make a phone call; lack of social activities; hearing impairment affecting daily activities; visual impairment affecting daily activities |

SpO_2_, peripheral oxygen saturation measured with a pulse oximeter device.
