# Supplementary figures and images for: Prediction of adverse health outcomes using an electronic frailty index among nonfrail and prefrail community elders
Source: BMC Geriatr. 2023 Aug 7;23:474. doi: 10.1186/s12877-023-04160-1 (PMC10408173; doi:10.1186/s12877-023-04160-1)

**Supplementary Figure.** The technique settings and assessment approaches in the study.

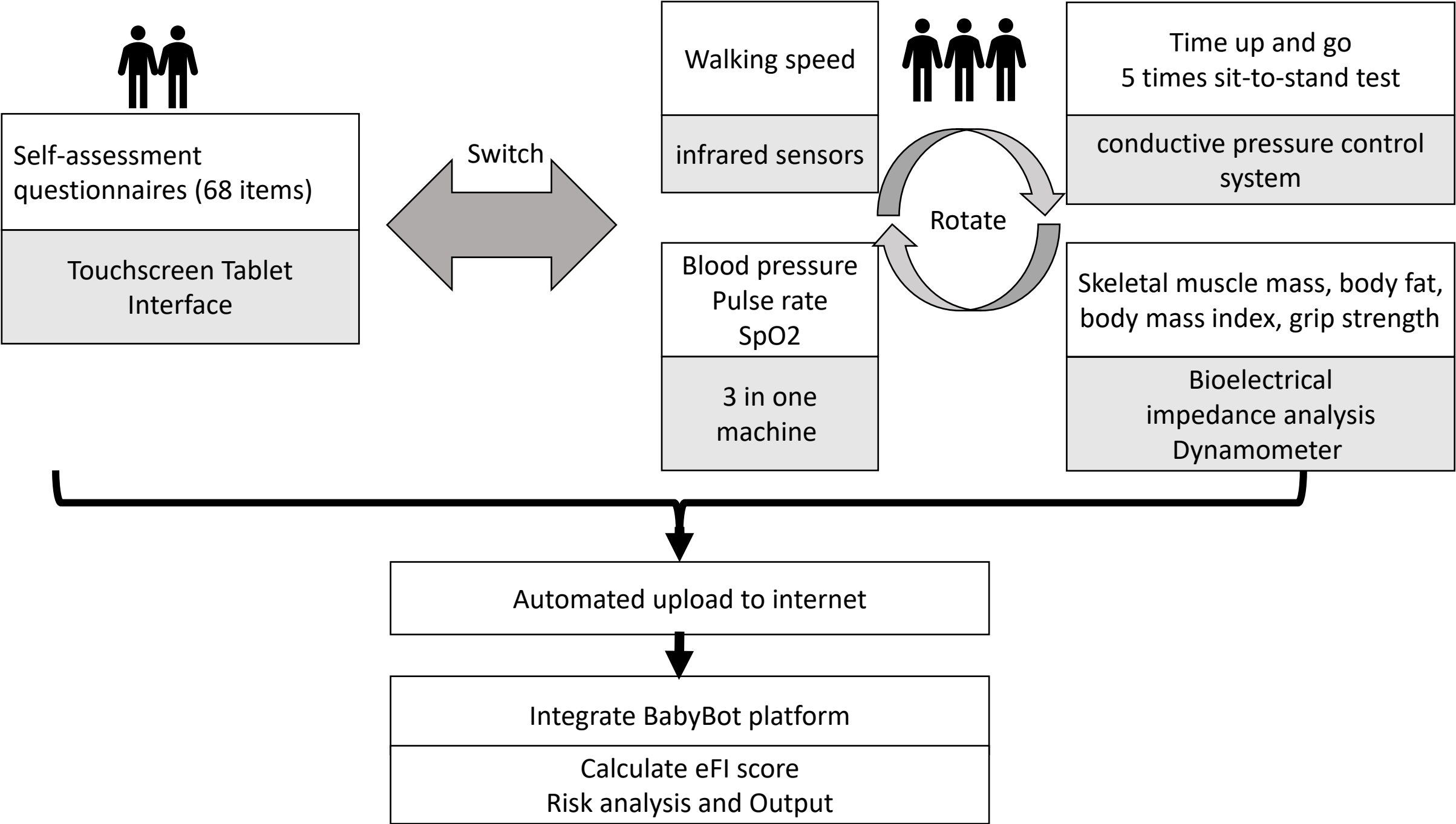

Supplement: Supplementary file 2 — Supplementary Material 2 [file 12877_2023_4160_MOESM2_ESM.pdf]
